# Schooling behavior driven complexities in a fear-induced prey–predator system with harvesting under deterministic and stochastic environments

**DOI:** 10.1038/s41598-023-28409-2

**Published:** 2023-01-22

**Authors:** Nazmul Sk, Samares Pal

**Affiliations:** Department of Mathematics, University of Kalyani, Kalyani, 741235 India

**Keywords:** Ecological modelling, Applied mathematics

## Abstract

The well-being of humans is closely linked to the well-being of species in any ecosystem, but the relationship between humans and nature has changed over time as societies have become more industrialized. In order to ensure the future of our ecosystems, we need to protect our planet’s biodiversity. In this work, a prey–predator model with fear dropping prey’s birth as well as death rates and nonlinear harvesting, is investigated. In addition, we consider that the consumption rate of predators, i.e., the functional response, is dependent on schooling behavior of both species. We have investigated the local stability of the equilibrium points and different types of bifurcations, such as transcritical, saddle-node, Hopf and Bogdanov–Takens (BT). We find that consumption rate of predator, fear and harvesting effort give complex dynamics in the neighbourhood of BT-points. Harvesting effort has both stabilizing and destabilizing effects. There is bistability between coexistence and predator-free equilibrium points in the system. Further, we have studied the deterministic model in fluctuating environment. Simulation results of stochastic system includes time series solutions of one simulation run and corresponding phase portraits. Notably, several simulation runs are conducted to obtain time series solutions, histograms, and stationary distributions. Our findings exhibit that during stochastic processes, model species fluctuate around some average values of the deterministic steady-state for lower environmental disturbances. However, higher values of environmental disturbances lead the species to extinction.

## Introduction

In order to shape the evolution of organisms, interspecies interactions are imperative. Over the last few decades, the scientific community has been studying prey–predator interactions extensively as one of the most exhibited examples of interspecies interactions. To understand the dynamics of prey–predator interactions on a global scale, numerous research studies have been conducted^1–6^. The functional response describes the mechanism by which predators feed on prey in prey–predator interactions, which is a central component of population dynamics. Different types of functional responses were developed in^7–10^. Functional responses from the Holling family are commonly used to describe prey–predator interactions^7^. But, the Holling family does not consider predator interference in the response function, whereas some biologists claim that predator interference must be included in the response function, particularly when predators compete for food or search for food^8,9,11^. The schooling behavior of predators and prey populations is explained by a functional response that is predator-dependent, which is outlined by Cosner et al.^10^. This type of behavior can be observed in biological species, such as a school of tuna searching and contacting herds of prey before hunting them^12^. Due to the assumption that predators were foraging collectively, the above functional response, unlike the others (e.g. ratio-dependent or Beddington-DeAngelis), increased with increasing predator density.

There are increasing numbers of evidences that predators impact their prey both directly (consumptive impacts) and indirectly (non-consumptive impacts). Directly, predators kill and consume their prey, but indirectly they instil fear in the prey population, which leads to significant behavioral changes in them (prey) such as vigilance, social behavior, habitat, foraging activity, reproductive cycle, etc.^3,13–15^. Due to this, prey species compensate with mortality risk, affecting their growth and mortality rates. Different prey–predator systems have been empirically observed to demonstrate such phenomena^16,17^. The cost of fear on prey reproduction is the main consideration in prey–predator models^4,18^. However, in reality, fear of predators can affect more than reproduction; it can also affect the death rates of prey population^5,19,20^. There is no doubt that harvesting is an influential issue from both an ecological and economic standpoint as well as from a social perspective. Moreover, there is a possibility that predators and/or prey species can exploit the ecosystem when there is an abundance of food. As a result, it is necessary to harvest species. There have been a number of researchers who have explored this area extensively in recent years^21–23^.

Stochasticity (i.e., variation in the model parameters due to random effects) in the population models improves the accuracy, realism, and utility of the models. Generally, population dynamics are affected by two types of stochasticity^24,25^. They are (i) Demographic stochasticity: random variation in the number of births and deaths in a population caused by the discrete nature of individuals. (ii) Environmental stochasticity: variability on the environmental conditions such as temperature, humidity, pH, rainfall, etc. It is obvious that survival and reproduction tend to be affected by environmental conditions. Environmental stochasticity applies to both small and large populations, whereas Demographic stochasticity negligible in case of large populations. Additionally, it is desirable to measure the variability of outcomes within a conservation or restoration framework^26^. To expose the actual dynamics of a population model within open environment, environmental stochasticity should always be considered as it is impossible to keep environmental conditions constant over time. Incorporation of environmental fluctuations or demographic stochasticity into the modeling approach are important components. In several existing literature^27^, author has shown that continuous fluctuation of environmental conditions can lead to random fluctuation in the important model parameters to a greater or lesser extent. They are mainly birth rates, death rates, carrying capacity, competition coefficients and all other parameters involved in the dynamical system. It has been established that environmental noise has a significant effect on deterministic systems when random disturbances are introduced^22,28–31^. Noise from the environment adversely affects almost all ecosystems. Thus, prey–predator models cannot ignore the shifting environmental effects, whereas stochastic models can accurately predict dynamics when the environment changes^22^. Belabbas et al.^32^ investigated a new approach of a stochastic prey–predator model with protection zone for the prey and found rich dynamics of the system.

Following the above discussions, we were motivated to visit the state as discussed here. There are hardly any studies considering predator-dependent functional response describing both predatory and prey schooling behaviors^10,33^. Additionally, a limited number of literatures address the issue of fear effect affecting the death of prey population^5,34,35^. Here, we intend to explore the deep insights of a prey–predator model with Cosner-type functional response^10^, fear that affects the growth and death of prey population, and nonlinear harvesting of the predator population. To the best of our knowledge, none yet studied the combined effects of double fear, nonlinear harvesting on Cosner-type functional response to fill the gap in extant research. Additionally, we incorporate white noise^36,37^ due to the perturbation of environmental conditions. Our second objective is to determine how environmental noise affects the dynamics of the system. Moreover, a numerical comparison between deterministic and stochastic models is made.

## Deterministic model

In a region under consideration, let at any instant $t>0$, *x* and *y* represent the prey and predator population densities, respectively. The rate of change of each model species density at time *t* is made on the following assumptions:

1. Prey population grow logistically in the absence of predator with birth rate *r*, which is affected by the fear ($f_1$) of predator (when predators are around).
2. There is a reduction in the rate of prey density change due to three types of death, namely, natural death with the rate $d_1$, fear related death^5^ with the level of fear $f_2$ and over crowding death with the rate $d_2$.
3. Also, the rate of change of prey density decreases due to predation of predator population following a predator-dependent functional response describing both predatory and prey schooling behaviors^10^. Response function is expressed in functional form describing as $\zeta (x, y)=\frac{cxy}{1+chxy}$, where *c* denotes the rate of consumption and *h* represents handling time of predator for one prey.
4. Predator population survive in the system by consuming prey population only. They grow with conversion efficiency $c_1$ of prey biomass into predator biomass.
5. Predator population harvested from the system which reduces its rate of density. We consider a nonlinear harvesting term (Michaelis-Menten type) given by, $H(y)=\dfrac{qEy}{p_1E+p_2y}$. Here, parameters *q* and *E*, respectively, represent the catchability rate and harvesting effort. It is easy to observe that $H\rightarrow \frac{q}{p_1}y$ as $E\rightarrow \infty$ for a fixed value of *y*. Also, $H\rightarrow \frac{q}{p_2}E$ as $y\rightarrow \infty$ for a fixed value of *E*. Therefore, at higher effort levels, $p_1$ is proportional to the stock level-catch rate ratio and at higher levels of stock, $p_2$ is proportional to the effort level-catch rate ratio.
6. Lastly, we assume that the predator population experience natural as well as over crowding related death with the rates $d_3$ and $d_4$, respectively.

Keeping all these above assumptions in mind, we formulate the following prey–predator model:

1 $$\begin{aligned} \frac{dx}{dt}= & {} \frac{rx}{1+f_1y}-(1+f_2y)d_1x-d_2x^2-\frac{cxy^2}{1+chxy}\nonumber ,\\ \frac{dy}{dt}= & {} \frac{c_1cxy^2}{1+chxy}-d_3y-d_4y^2-\frac{qEy}{p_1E+p_2y}. \end{aligned}$$

System (1) is to be analyzed with the initial conditions $x(0),y(0)>0$. All the model parameters are assumed to be positive constants and their hypothetical values that we used for numerical calculations are as follows:

2 $$\begin{aligned}{} & {} r=3.1, \ f_1=1, \ f_2=0.4, \ d_1=0.1, \ d_2=0.08, \ c=0.11, \ h=0.1, \ c_1=0.5, \ d_3=0.1,\nonumber \\{} & {} d_4=0.06, \ q=0.65, \ E=0.5, \ p_1=0.5, \ p_2=0.65. \end{aligned}$$

**Figure 1:**
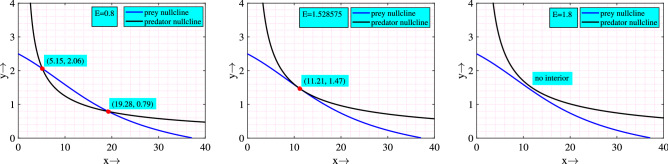
Nullclines for different values of *E*. Other parameters are same as in (2).

In Table 1, we have provided system’s equilibria, sufficient conditions of their existence and stability. Mathematically, it is difficult to determine the existence of coexistence (interior) equilibrium point(s) by the given nullclines. So, we visualize it numerically (see Fig. 1). It is apparent from the figure that on increasing the value of *E*, number of coexistence equilibrium points reduces and after a certain range there is no coexistence equilibrium point.

**Table 1:** Sufficient conditions for the existence and stability of different equilibrium points of system (1).

Equilibria	Condition of existence	Stability condition
$E_0=(0,0,0)$	Always exist	$r<d_1$
$E_1=\left( \dfrac{r-d_1}{d_2},0\right)$	$r>d_1$	$r>d_1$
$E^*=(x^*,y^*)$	$x^*$ and $y^*$ are the positive solution(s) of the nullclines $\frac{r}{1+f_1y}-(1+f_2y)d_1-d_2x-\frac{cy^2}{1+chxy}=0$ and $\frac{c_1cxy}{1+chxy}-d_3-d_4y-\frac{qE}{p_1E+p_2y}=0.$	$B_1>0, \ B_0>0, \ B_1=-(b_{11}+b_{22})$, $B_0=b_{11}b_{22}-b_{12}b_{21}, \ [b_{ij}]_{2\times 2}=J_{E^*}$

### Transcritical bifurcation

From Table 1, it is clear that the equilibrium $E_0$ is stable if $r<d_1$, which is opposite to the existence condition of $E_1$. That is, equilibrium $E_0$ is stable whenever the equilibrium $E_1$ does not exist, and hence these two equilibria are related via transcritical bifurcation. Using Sotomayor theorem^38^, we can easily prove that model system (1) experiences transcritical bifurcation at the trivial equilibrium point $E_0$ as the growth rate of prey crosses a critical value $r^{[TB]}=d_1$.

**Figure 2:**
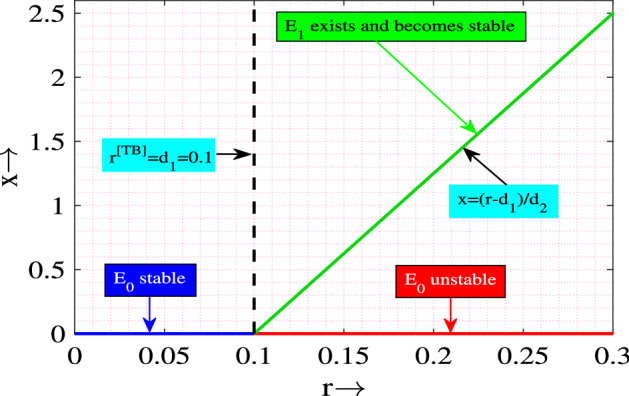
Transcritical bifurcation with respect to *r*. Rest of the parameters are same as in (2).

We visualize the transcritical bifurcation graphically in Fig. 2. It is clear from the figure that when the value of *r* is less than $d_1=0.1$, the equilibrium point $E_0$ only exists and it is stable. If we increase the value of *r* ($r>r^{TB}=d_1=0.1$) then $E_0$ becomes unstable and the equilibrium point $E_1$ exists and becomes stable.

### Hopf bifurcation

One of the most common dynamics in interacting population dynamics is oscillating behavior, which implies that there is a Hopf bifurcation. By local changes in equilibrium properties, Hopf bifurcation describes when a periodic solution appears or disappears. In this section, we study the Hopf bifurcation through the coexistence equilibrium $E^*$ with respect to the model parameter *E*. Discussion for the existence of Hopf bifurcation is as follows:

As it is easy to follow, we verify Hopf bifurcation numerically. We have considered the parameters value same as in (2) except $c=0.1$ and *E*. At $E=E^{[HB]}=0.1196559641$, the trace of the Jacobian matrix at $E^*(2.618402886, 2.352228027)$ is zero and determinant, $Det(J_{E^*})=0.4474794791>0$. The value of $\dfrac{d(Tr(J_{E^*}))}{dE}\Big |_{E=E^{[HB]}}=-0.02965188514\ne 0$. Therefore, the transversality condition for Hopf bifurcation is also satisfied at $E=E^{[HB]}$. Thus, these results confirm that the system (1) experiences a Hopf bifurcation^2^ around $E^*(2.618402886, 2.352228027)$.

Moreover, we obtain Lyapunov number $L_1=-0.04728284756\pi <0$ at $E^{[HB]}=0.1196559641$. This implies that system (1) goes through a supercritical Hopf bifurcation at $E^*(2.618402886, 2.352228027)$.

### Saddle-node bifurcation

Sotomayor’s theorem says that saddle-node bifurcation may occur at coincident equilibrium points depending on threshold value of the bifurcation parameters. Since the analytical result is difficult to follow, we verify the existence of saddle-node bifurcation of system (1) numerically for given set of parameters value (2) except $c=0.15$ and *E*. At $E=E^{[SN]}=2.6670276565$, system (1) has a coincident equilibrium $E^*(12.07536421, 1.327137944)$. The corresponding Jacobian matrix $$J_{E^*}(E=E^{[SN]})= \left( \begin{matrix} -0.9247512109 &{} -10.89563235\\ 0.08585790896 &{} 1.011635174 \end{matrix} \right)$$ has one zero eigenvalue. For the matrices $J_{E^*}$ and $J_{E^*}^T$ , the eigenvectors *V* and *W* associated with the zero eigenvalue can be found as follows: $V=[-7.625518027 \quad 0.6469271584]^T$ and $W=[-0.06805575397 \quad -0.7326946399]^T$. This yields,

$$\begin{aligned} W^TF_E\left( E^*, E^{[SN]}\right) =0.1130463331\ne 0; \ W^TD^2F\left( E^*,E^{[SN]}\right) (V,V)=-0.3491755781\ne 0. \end{aligned}$$

Based on Sotomayor’s theorem^38^, we can conclude that system (1) experiences a saddle-node bifurcation at the equilibrium point $E^*$ when the parameter *E* crosses the critical value $E=E^{[SN]}=2.6670276565$.

### Bogdanov–Takens bifurcation

A number of one-dimensional bifurcations such as transcritical, Hopf and saddle-node have been studied in earlier segments. Each of these bifurcations belongs to one parametric bifurcation. In the present segment, we will discuss two parametric bifurcation, i.e., of codimension two bifurcation. Bogdanov–Takens bifurcation (BT) is a such type of bifurcation. When Hopf and saddle-node bifurcation curves collide, a bifurcation of this type occurs at the vicinity of colliding point. In this case, the critical values of two bifurcation parameters give zero eigenvalues of multiplicity two in the Jacobian matrix of the system.

One can used Kuznetsov’s^39^ technique to achieve the standard form of BT-bifurcation. Due to model complexity, we did not find any explicit expression of BT-bifurcation. So, we verify it numerically, which is discussed below.

System (1) undergoes BT-bifurcation for the set of parameters given in (2) except *c* and *E*. We consider *c* and *E* as bifurcation parameters and observe that saddle-node and Hopf bifurcation curves collide with each other at $(E, c)=(1.42829255, 0.105462715)=\left( E^{[BT_1]}, c^{[BT_1]}\right)\,$ and $\,(E, c)=(7.345497850, 0.2137076372)=\left( E^{[BT_2]}, c^{[BT_2]}\right)$, and the instantaneous equilibrium points are (11.12253608, 1.485272279) and (13.778434, 1.13304881), respectively. Therefore, system (1) undergoes through two BT-bifurcation points: one for $E^*=(11.122536, 1.4852723)$ at $\left( E^{[BT_1]}, c^{[BT_1]}\right) =(1.42829255, 0.105462715)$ and another for $E^*=(13.778434, 1.13304881)$ at $\left( E^{[BT_2]}, c^{[BT_2]}\right) =(7.345, 0.213)$.

### Numerical results of the deterministic system (1)

To explore the rich dynamics of the system (1), we perform extensive numerical simulations in this section. For numerical simulations, we choose a set of biologically feasible hypothetical parameter values as given in (2).

**Figure 3:**
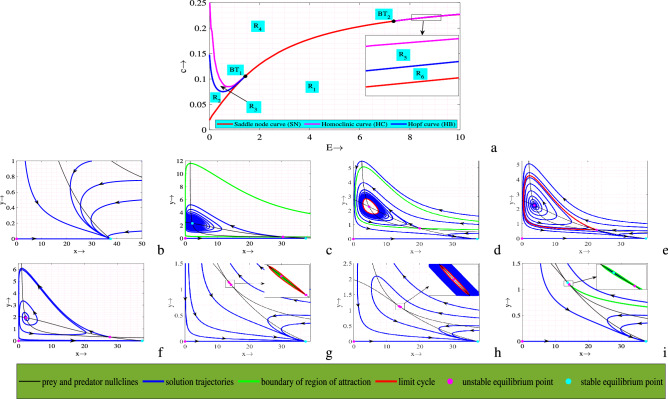
In the figures, (**a**) Bifurcation diagram of system (1) in $E$ $-$ $c$ plane. Rest are full phase portraits in regions (**b**) $R_1$, (**c**) $R_2$, (**d**) $R_3$, (**e**) on HC, (**f**) $R_4$, (**g**) on HC, (**h**) $R_5$ and (**i**) $R_6$. Parameters are at the same values as in (2) except *c* and *E*.

A system may exhibit rich dynamical behavior if its dynamics are investigated in different bi-parameter spaces. So, here we plot two bi-parametric bifurcations (Figs. 3a, 5a). In Fig. 3a, we plot saddle-node, Hopf and Bogdanov–Takens bifurcations in $E$ $-$ $c$ plane, which divide the entire region into six different regions ($R_1$ $-$ $R_6$) of different dynamical behaviors. Complete phase portraits are drawn for every region in order to understand their dynamics transparently, Fig. 3b–i. It is apparent from Fig. 3a that the saddle-node curve divides the whole region into two parts. Among these two parts one part is region $R_1$, which has no interior equilibrium point (see Fig. 3b) and other part is sum of rest regions ($R_2$ $-$ $R_6$), that contain two interior equilibrium points (see Fig. 3c–i). Next, for lower and higher values of *E*, there exist Hopf bifurcation curves, which divide two portions of the region into two subregions that are ($R_2$/$R_3$) and ($R_5$/$R_6$). In one side of the Hopf curve, one of the two interiors is stable spiral and other one is saddle, Fig. 3c,i (correspond to the regions $R_2$ & $R_6$, respectively). On the other hand, as the value of *E* and *c* crosses Hopf curve, stable spiral point becomes unstable spiral (stable limit cycle) and saddle one remain same, Fig. 3d,h (correspond to the regions $R_3$ & $R_5$, respectively). Similar as Hopf curve, there exist two Homoclinic curves for lower and higher values of *E*, which divide a subregion into two another subregions ($R_3$/$R_4$) and ($R_4$/$R_5$). As the values of (*E*, *c*) closest to the Homoclinic curve system gives larger amplitude-limit cycle and on the curve it gives maximum amplitude-limit cycle, Fig. 3e,g. Further, if the values of (*E*, *c*) crosses the Homoclinic curve, stable limit cycle destroys and the equilibrium point becomes unstable (see Fig. 3f corresponds to the region $R_4$). Also, it is clear from Fig. 3a that there exist two BT-points ($BT_1=(1.428292550, 0.1054627152)$ and $BT_2=(7.345497850, 0.2137076372)$), where aforementioned three bifurcation curves meet with each other. Therefore, the neighbourhood of BT-points exhibits complex dynamics of the system.

**Figure 4:**
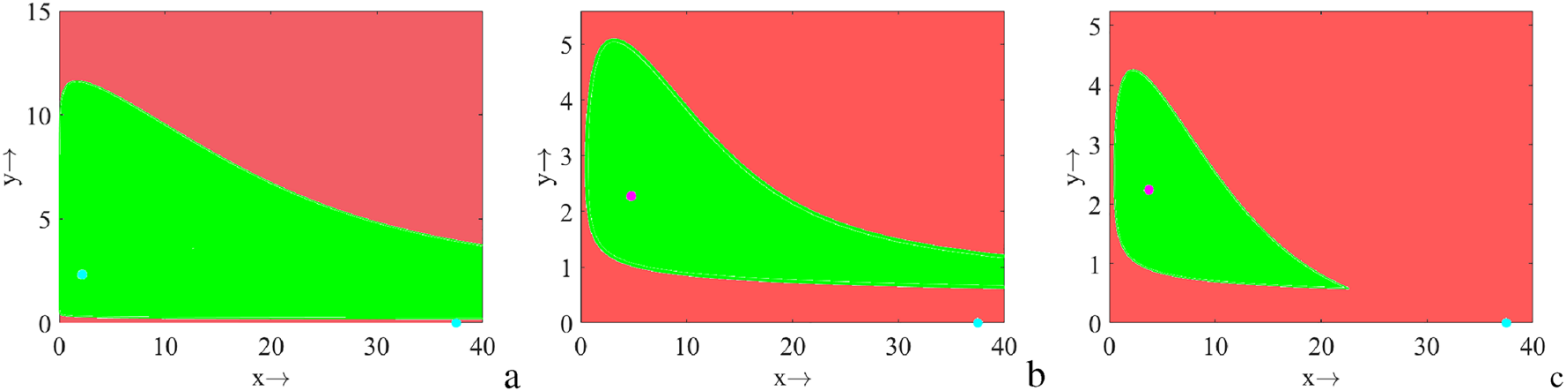
Regions of attraction of the equilibria $E^*$ (green regions) and $E_1$ (red regions) for the regions (**a**) *R*_2_, (**b**) *R*_3_, and (**c**) on HC.

**Figure 5:**
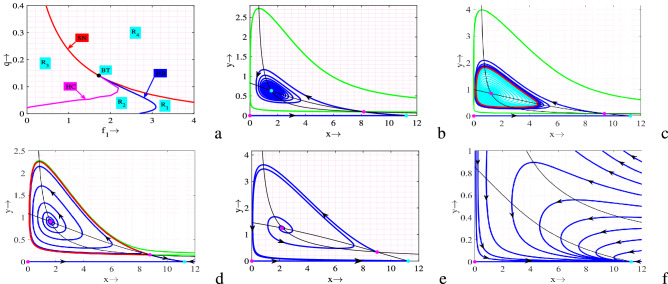
Bifurcation diagram of system (1) in (a) $f_1$ $-$ $q$ plane and the corresponding full phase portraits of different subregions. Phase portraits in regions (**b**) $R_1$, (**c**) $R_2$, (**d**) on HC, (**e**) $R_3$ and (**f**) $R_4$. Parameters are at the same values as in (2) except $a=1$, $c=0.2$, $c_1=0.8$, $E=2.5$, $f_1$ and *q*.

It is observed that the system preserves bistability for the regions $R_2$, $R_3$, $R_5$ and $R_6$. For a fixed set of parameter values, depending on the initial population size the trajectories initiating inside the green regions converge to the stable interior (stable limit cycle) whereas the trajectories initiating in the red regions converge to the predator-free equilibrium point, Fig. 4. In Fig. 5a, we plot another bi-parametric bifurcation in $f_1$ $-$ $q$ plane, which is divided into four subregions ($R_1$ $-$ $R_4$) of different dynamics by different bifurcation curves. Phase portraits of every region are plotted (see Fig. 5b–f). We observe that there is no interior for diagonally higher values of both of the parameters $f_1$ and *q* whereas two interiors exist when the values of $f_1$ and/or *q* are lower, Fig. 5a. Detailed discussions of the figure are same as previous one (Fig. 3).

**Figure 6:**
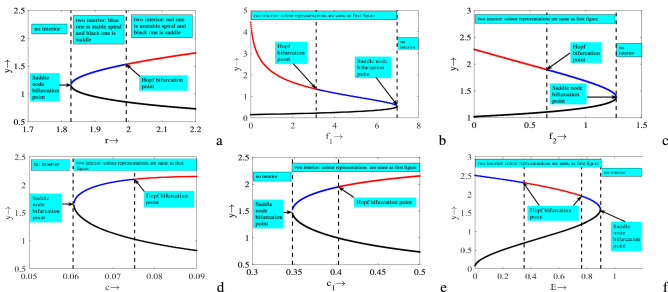
Bifurcation diagrams of system (1) with respect to (**a**) *r*, (**b**) $f_1$, (**c**) $f_2$, (**d**) *c*, (**e**) $c_1$ and (**f**) *E*. Rest of the parameters are at the same values as in (2) except in (**a**) $c=0.1$, $E=0.3$; (**b**) $c=0.15$, $E=0.06$; (**c**) $c=0.08$, $E=0.7$; (**d**) $E=0.6$; (**e**) $c=0.1$, $E=0.6$; (**f**) $c=0.0782$.

Next, to observe the effect of important model parameters explicitly, we plot one parameter bifurcation diagrams while other parameters are fixed, Fig. 6. It is clear form Fig. 6a,d,e that for lower values of the parameters *r*, *c* and $c_1$, there is no interior. As the value of these parameters surpasses saddle-node bifurcation point there exist two interior one of which always remain saddle and another one switches its stability (stable spiral $\Rightarrow$ unstable spiral) through Hopf bifurcation point. Therefore, up to a certain range of these parameters have destabilizing effect. On the other hand, for lower values of the parameters $f_1$, $f_2$ and *E* there exist two interior one of them is always saddle and another one switches its stability (unstable spiral $\Rightarrow$ stable spiral) through Hopf bifurcation point (see Fig. 6b,c,f). But, when the value of these parameters crosses saddle-node bifurcation point, no interior equilibrium point exists. Note that up to a certain range of these parameters, $f_1$ and $f_2$ have stabilizing effect whereas *E* has both destabilizing as well as stabilizing effects.

### Interpretation of the deterministic results in the context of biology

For the deterministic system, we mainly investigate different types of bifurcation results. They are Transcritical, Hopf, Saddle-node and Bogdanov–Takens. In the context of a biological system, a bifurcation occurs when a small smooth change made to the parameter values (the bifurcation parameters) of a system causes a sudden ’qualitative’ or topological change in the behavior of the system. Bifurcations mainly describe changes in the stability and/or existence of fixed points (equilibrium points) and the bifurcation parameters act as a control parameter. Changes in the control parameter eventually changed the qualitative behavior of the system. Above mentioned bifurcations of the considered system (1) were found for the model parameters *r*, $f_1$, $f_2$, *c*, $c_1$, *q* and *E*. Therefore, all these ecological parameters (i.e., growth rate of prey, fear, capture rate, harvesting effort) act as control parameters of the proposed system. In Transcritical bifurcation, there is a critical value of the bifurcation parameter from which two equilibrium points exchange their stability. Moreover, on the one side of the critical value one of the two equilibrium points exist while on the other side both equilibrium points exist. Therefore, we have a critical value of an ecological parameter from which species persistency and stability can be described. In case of Hopf bifurcation, there is a critical value of a parameter from which densities of model species either fluctuate from fixed stable densities or vice versa. In the case of Saddle-node bifurcation, coexistence equilibrium points exist or diminish in pair by varying bifurcation parameter i.e., from the critical value of the bifurcation parameter coexistence equilibrium points exists or diminish in pair. Lastly, BT-bifurcation, the point where saddle-node curve and Hopf curve meet is known as BT-point. Thus, all these phenomena occur around the BT-point, i.e., near the BT-point complex dynamics (like species persistency, stability, extinction) occurs. Thus, studying the bifurcation in prey–predator systems has great importance from environmental perspective. In order to maintain an ecological balance between prey and predator populations, identification of bifurcation parameters plays a crucial role in determining an effective control strategy.

## Stochastic model

There is no doubt that the system (1) is derived based on the assumption that all the input variables follow deterministic laws and are deterministic functions of time. However, in mathematical modeling of ecosystems, the deterministic system has its limitations since it cannot capture the influence of random environmental fluctuations in its parameters^31^. In order to study the effects of environmental fluctuations on the entire ecosystem, it is reasonable to introduce the noise term into the deterministic model. Mao et al.^40^ demonstrated that one or more system parameter(s) can be perturbed stochastically with white noise term to derive stochastic system. Note that the approach to formulate the stochastic model based upon existing deterministic model is not unique^41,42^. Introducing multiplicative noise terms into the growth equations of both prey and predator populations, we formulate the following stochastic model.

3 $$\begin{aligned} dx= & {} \left[ \frac{rx}{1+f_1y}-(1+f_2y)d_1x-d_2x^2-\frac{cxy^2}{1+chxy}\right] dt+\sigma _1 xdB_1(t)\nonumber ,\\ dy= & {} \left[ \frac{c_1cxy^2}{1+chxy}-d_3y-d_4y^2-\frac{qEy}{p_1E+p_2y}\right] dt+\sigma _2 ydB_2(t), \end{aligned}$$

where $\sigma _i$ $(i=1, 2)$ represent the intensity of environmental fluctuations and $B_i(t)$ are standard Brownian motions.

Throughout the analysis, we take $(\Omega ,\mathcal {F},\mathcal {P})$ as a complete probability space with a filtration $\{\mathcal {F}_t\}_{t\in \mathbb {R}_+}$ satisfying the conventional condition, namely right continuity and increasing, whereas $\mathcal {F}_0$ consists of all $\mathcal {P}$ $-$void sets^40^. Any solution of system (3) subjected to the positive initial condition is an It$\hat{\text {o}}$ process^43^. Without any loss of generality we assume $\sigma _1,\sigma _2>0$.

### Numerical results of stochastic system (3)

In this section, we simulate the stochastic model (3) to explore the dynamics of different noise intensities.

**Figure 7:**
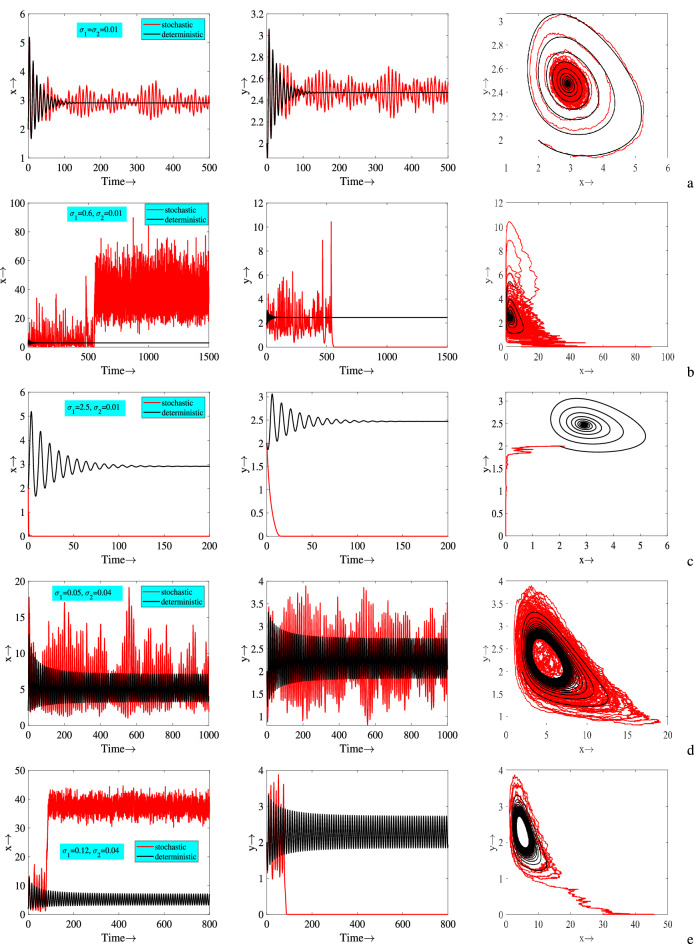
Time series solutions of systems (1) and (3) and corresponding phase portraits for different values of $\sigma _1$ and $\sigma _2$. Parameters are at the same values as in (2) except in (**a**–**c**) $c=0.08$, $E=0.06$ and in (**d**,**e**) $c=0.077$, $E=0.4$.

In Fig. 7, we plot deterministic and stochastic time series solutions and their corresponding phase portraits. Random perturbations in the model parameters (i.e., environmental noise) destroy the stability of deterministic equilibrium and lead to weak stochastic stability known as stationary distributions. According to Fig. 7a, in the absence of environmental noise, model variables approach to their equilibrium values, and in the presence of environmental noise, stationary distributions are observed. Our results indicate that when noise intensity is relatively low, the stochastic system still maintains some stability. Now, if we increase the value of $\sigma _1$ to 0.6 from 0.01, then after some initial transients predator population extinct. However, it is important to note that in this case the prey species is not fluctuating around the deterministic steady-state rather it fluctuate above its equilibrium value (obviously, it happen due to predator extinction) (see Fig. 7b). Next, we choose $\sigma _1=2.5$ and $\sigma _2=.01$, in this case both the model species go to extinction, Fig. 7c. Moreover, it is clear from the figure that prey species goes to extinction first, before predators. However, the model species approach to their equilibrium values in the absence of environmental noise. It is important to note that in all of these instances of species extinction, extinction time may vary from simulation to simulation, but extinction is confirmed in every simulation. Lastly, we fixed our deterministic system in oscillatory state and see the effect of environmental disturbances. We observe that lower strength of environmental noise propel the species to fluctuate around the limit cycle (see Fig. 7d). However, higher strength of disturbances (i.e., environmental noise) ultimately lead predator population to extinction after some initial transient dynamics (see Fig. 7e). In this case, fluctuations of prey density occur above the deterministic density.

**Figure 8:**
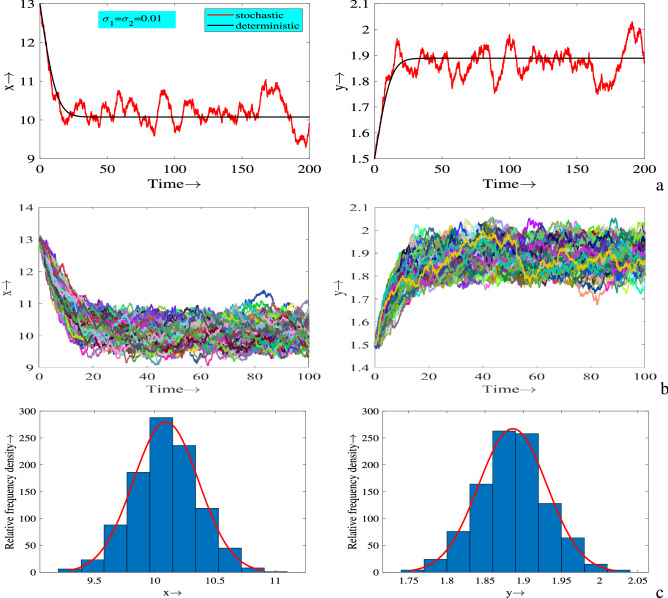
(**a**) Time series solutions of systems (1) and (3); (**b**) solution trajectories of *x* and *y* for 200 simulations; (**c**) relative frequency density of prey and predator populations for 1000 simulations. Parameters are at the same values as in (2) except $c=0.027$, $E=0.06$ and $\sigma _1=\sigma _2=0.01$.

To be more transparent of the effect of stochasticity, 200 simulations are plotted in Fig. 8 as time series solutions. It can be easily observed that all the solution trajectories fluctuate around the deterministic steady-state due to environmental disturbances, Fig. 8b. Histogram plots of 1000 simulation runs also show that these fluctuations are observed at the stationary distributions, where prey population is distributed within the range (9, 11) and predator population within the range (1.75, 2) (see Fig. 8c).

**Figure 9:**
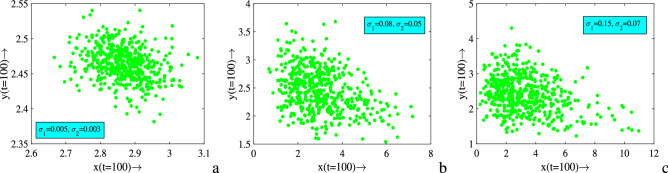
Stationary distributions of prey (*x*) and predator (*y*) populations around deterministic steady-state for different values of $\sigma _1$ and $\sigma _2$. Parameters are at the same values as in (2) except $c=0.08$, $E=0.06$.

Population fluctuation due to environmental noise is also reflected in the stationary distributions. Therefore, for better presentation and understanding the stochastic effect on population dynamics, stationary distributions of different noise intensities are attained. The result of stationary distributions obtained from 500 simulations at $t=100$, which is presented in Fig. 9. We observe that the population distributions occur in wider range as the noise intensity increases. Whenever the strength of environmental disturbances is low, prey population are distributed within (2.6, 3.1) and it is estimated that the predator population are within the range of (2.35, 2.55). In the next case they are within (0, 8) and (1.5, 4), respectively for moderate strength of noise. Lastly, for higher strength they are within (0, 12) and (1,5), respectively. In these cases, there are no extinction scenarios associated with these parameters, since they do not satisfy the conditions of extinction. Thus, we conclude from these results that the strength of environmental noise determines the amplitude of oscillation and average value of populations at all future times.

### Interpretation of the stochastic results in the context of biology

Maximum models assume a deterministic, unchanging environment, whereas real environments are uncertain and stochastic. Elton^44^ observed that the ”chief cause of fluctuations in animal numbers is the instability of the environment. The climate in most countries is always varying. . . .” Therefore, stochastic models are preferred over deterministic ones. From numerical results we observe that if the intensity of environmental noise is small, prey and predator populations will survive for a long time. Therefore, intensities of environmental noise are conducive to the survival of the population. Also, it is evident that there is an important role of environmental noises for the persistence of prey and predator populations weekly as well as strongly. We notice that the prey population can persist for low intensity of noise, higher growth rate of prey, lower level of both fears and consumption rate. The predator population can persist for higher values of conversion efficiency of predator and lower values of catching capability and low intensity of noise on predator population. Population extinction is a serious issue from an ecological perspective. It is observed that high intensity of noise plays an crucial role in extinction of the species. The extinction of prey species drive predator species towards extinction as they depend on prey species only. Further, under a circumstances when the environmental disturbance for prey is low while for predator is high, only predator population extinct. Furthermore, at the simulation time, we notice that increasing intensity of noise decreases average extinction time. More importantly, chaotic variation in the population size due to environmental fluctuations have serious implication, it reduces species extinction^45^. These findings are consistent with the reality which imply that in any natural ecosystem, environmental fluctuations have significant influences on the persistence and extinction of interacting species. As a result, a conservationist may be able to take a variety of measures to prevent species extinctions by identifying different routes to extinction.

## Discussion and conclusion

We propose a prey–predator model where schooling behaviour of both prey and predator are considered by a predator-dependent functional response. Predator-induced fear is assumed to affect the birth as well as death rates of the prey population. In order to conserve resources and manage the social environment, predator population are harvested. The complete investigation or discussion of the system is mainly devoted to the important ecological factors, namely, the fear factors, consumption rate of predator (which depend on schooling behavior of the species) and harvesting. Firstly, we study the dynamics of the deterministic system and then stochastic system. Both these systems were studied in detail. For deterministic system, we have established the existence and stability conditions of the equilibrium points. Different types of bifurcations are also numerically investigated, including transcritical, saddle-node, Hopf, and Bogdanov–Takens bifurcations. We have plotted two parameter bifurcation diagrams in which the aforementioned bifurcation curves are plotted which divide the whole region into subregions of different dynamics. Complex dynamics are observed around the BT-points for harvesting effort, fear, and predator consumption rate. We find that growth rate of prey, consumption rate of predator and conversion efficiency of prey biomass into predator biomass have destabilizing effects. In contrast, fear affecting growth and death of prey has stabilizing effects. The harvesting effort, however, has both stabilizing and destabilizing effects. Further, for lower values of predator consumption and higher values of harvesting effort, there is no interior (coexistence) equilibrium point. A BT-point has been observed around a low harvesting effort and moderate consumption rate, exhibiting complex dynamical behavior around it (BT-point). Therefore, these parameter values compensate with each other to exhibit complex biological dynamics. Moreover, moderate values of both fear affecting prey’s growth and catching capability enrich the system with rich biological dynamics around the BT-point. The predator population is at risk of extinction due to higher harvesting effort and lower consumption rates. Higher levels of both fear and catching capability also eliminate predator populations from ecosystems. Both species, however, coexist and switch between stability for lower and higher levels of harvesting effort. Prey and predator populations always live in harmony at lower values of catching capability, regardless of the value of fear dropping prey’s birth. As the amount of fear increases, coexisting species change their stability from unstable spiral to stable spiral.

Furthermore, we incorporate multiplicative noise terms in the deterministic system to understand the dynamics in the presence of environmental driving forces. We can obtain that there exists a unique positive global solution for the stochastic model and determined the conditions under which a species is likely to persist or fail. We did not include formal mathematical analysis of these results. According to our numerical findings, species survival is closely linked to the intensity of environmental fluctuations. Recently, Rogers et al.^46^ showed that “Chaos is not rare in natural ecosystems”. Therefore, chaotic nature in the population densities due to environmental fluctuations is an important result. Biologically, chaotic variation in the population size have serious implication: it has the ability to reduce species extinction^45^. Moreover, we can obtain parametric conditions mathematically under which stationary distributions exist in the stochastic system. According to simulation results, these parametric restrictions will not hold for large-amplitude environmental noise. Consequently, it can destabilize the system, and, in that case, no stationary distribution can be found^32,47^. As a result of the existence of stationary distributions, there can be some degree of stochastic stability. In terms of biology, this indicates that both prey and predator populations coexist on a long-term basis, leading to the conclusion that the system is permanent. Based on numerical results of the stochastic system, we have demonstrated that for lower levels of noise, stationary distributions are attained, while high levels of noise lead to species extinction. It is possible to observe that there are two different scenarios of extinction: the first case is the both populations extinct; second case is the only prey population persist while predator extinct. From an ecological viewpoint, comparing the stochastic results with corresponding deterministic result, we observe two interesting facts. For low intensity of environmental fluctuations both prey and predator populations coexist in the long run, i.e., the system is permanent. Another fact is that none of the important ecological factor (growth rate of prey, fear, harvesting, consumption rate of predator) can avoid the extinction of model species when the nature exhibits large amount of environmental fluctuations. Although, all these important ecological factors have significant impact on species persistence and extinction in constant environment (i.e., deterministic environment). Following articles^48–50^, we can also define stochastic Hopf bifurcation^48,50^ and dynamic bifurcation^49^ as similar phenomena also happen in the considered system. Study of bifurcations in the stochastic framework is an interesting topic. In the near future, we will discuss this topic in more detail.

### Human or animal involvement

 The current study did not involve any animal or human experiments.

## Data Availability

All data generated or analyzed during this study are included in this article. The softwares used in this study are Maple (version-2019) and MATLAB (version-R2019a).
